# Supplementary material for: Exogenous Rubella Virus Capsid Proteins Enhance Virus Genome Replication
Source: Pathogens. 2022 Jun 14;11(6):683. doi: 10.3390/pathogens11060683 (PMC9228353; doi:10.3390/pathogens11060683)
Supplement: Supplementary file 1 [file pathogens-11-00683-s001.zip › pathogens-1722010-supplementary.pdf]

**Supplementary Materials:**

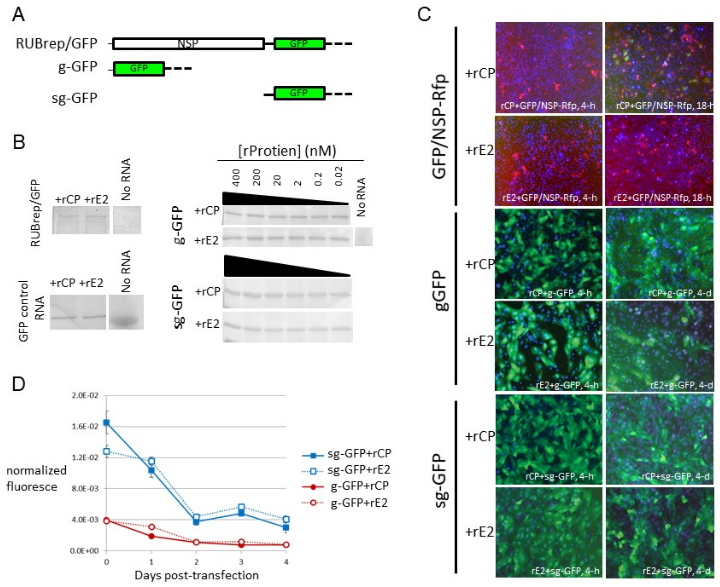


**Figure S1**. Effect of RuV rCP on translation of RNA *in vitro* (B) and in transient expression (C). (A) Schematic representation of RUBrep/GFP and RuV mini-Xpress systems. The g-GFP has the GFP gene inserted immediately downstream of the 5′ untranslated region (UTR), while the sg-GFP contains the sequence of RUBrep/GFP from nt 6436 to the 3′ terminus. All replicons maintained the RuV 3′ terminal 400 nts. The size of each fragment is not drawn to scale. (B) In *vitro* translation from different RNA templates with rCP or rE2 as indicated. Right: translation from RUBrep/GFP or pCI-GFP with 200 nM of rCP or rE2. Left: translation from the mini-Xpress system with serially diluted rCP or rE2. Images from the SDS polyacrylamide gel are shown. (-): no template control. (C) Live images of RFP or GFP expression in cells transfected with a RuV full-length replicon (NSP-RFP) or RuV mini-Xpress systems (g-GFP and sg-GFP) with rCP or rE2, as indicated. Cell nuclei were stained with Hoechst 33258. The images were taken at 4-h (left), 18-h (NSP-RFP; right) and 4-d (mini-Xpress; right) post-transfection using the Axiovert 200 at 100x. (D) Intracellular GFP expression from the transfection with g-GFP RNA, sg-GFP RNA, or pCI-GFP DNA with 100 ng of rCP or rE2 over 7 days of post-transfection (*X*-axis). Intracellular fluorescence was measured using Fluoroskan Ascent Microplate Fluorometer (Thermo Fisher Scientific). At each timepoint, GFP fluorescence was normalized to the Hoechst 33258 fluorescence (*Y*-axis). The data were collected from three independent transfections in triplicates.

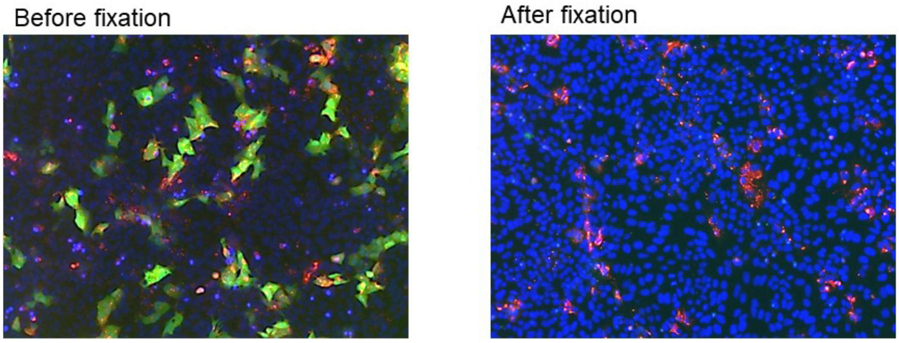


**Figure S2**. GFP expression in RuV GFP replicon-transfected cells before (left; live image) and after methanol fixation (right). Vero cells were transfected with RUBrep/GFP/NSP-Rfp RNA with 100 ng rCP. Cell nuclei were stained with Hoechst 33258. Images were taken from transfected cells at 3-d post-transfection at 100x by Axiovert 200.

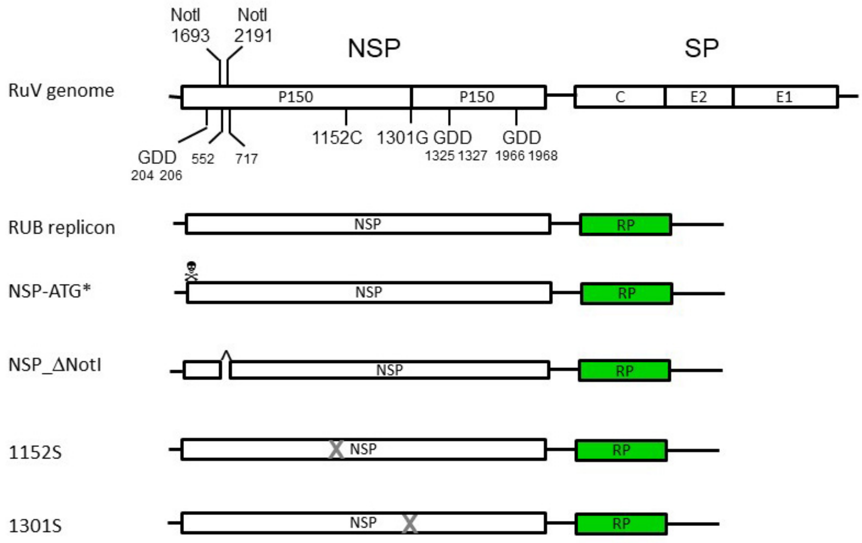


**Figure S3.** Schematic representation of RuV genome and replicons. The two ORFs, NSP and SP, in the RuV genome are denoted at the top. The size of each fragment is not drawn to scale. In RuV genome diagram, nucleotide positions of the two NotI sites in the NSP ORF are given at the top while the amino acid positions of the domains targeted for mutagenesis are listed at the bottom. The positions are denoted based on the sequence of F-therien strain (GenBank Accession Number NC_001545). RP: reporter gene.
